# RECENT ECOLOGICAL DIVERGENCE DESPITE MIGRATION IN SOCKEYE SALMON (*ONCORHYNCHUS NERKA*)

**DOI:** 10.1111/j.1558-5646.2009.00927.x

**Published:** 2010-06

**Authors:** Scott A Pavey, Jennifer L Nielsen, Troy R Hamon

**Affiliations:** 1 Biological Sciences Department, Simon Fraser UniversityBurnaby, British Columbia V5A 1S6 Canada; 2 National Park ServiceP.O. Box 7, King Salmon, Alaska 99613; 3 E-mail: spavey@sfu.ca; 4 U.S. Geological Survey, Alaska Science Center4210 University Dr., Anchorage, Alaska 99508

**Keywords:** Colonization, divergence with migration, ecological speciation, isolation with migration (IM), rapid evolution, sympatric speciation

## Abstract

Ecological divergence may result when populations experience different selection regimes, but there is considerable discussion about the role of migration at the beginning stages of divergence before reproductive isolating mechanisms have evolved. However, detection of past migration is difficult in current populations and tools to differentiate genetic similarities due to migration versus recent common ancestry are only recently available. Using past volcanic eruption times as a framework, we combine morphological analyses of traits important to reproduction with a coalescent-based genetic analysis of two proximate sockeye salmon (*Oncorhynchus nerka*) populations. We find that this is the most recent (∼500 years, 100 generations) natural ecological divergence recorded in a fish species, and report that this divergence is occurring despite migration. Although studies of fish divergence following the retreat of glaciers (10,000–15,000 years ago) have contributed extensively to our understanding of speciation, the Aniakchak system of sockeye salmon provides a rare example of the initial stages of ecological divergence following natural colonization. Our results show that even in the face of continued migration, populations may diverge in the absence of a physical barrier.

Populations subjected to different selection regimes can evolve reproductive isolation (Mayr 1947). This divergence ultimately may result in speciation arising from ecological differences (Bush 1994; Schluter 1996a,b, 2000; Feder et al. 2005; Rundle and Nosil 2005; Funk et al. 2006). In many cases of ecological divergence, a physical barrier to migration separates the populations in the initial stages (Schluter 2001; Schluter et al. 2001; Rundle and Nosil 2005). When the populations regain contact, isolating mechanisms (behavioral, morphological, etc.) have already evolved in the absence of migration. In populations that are currently sympatric, this may have occurred via a double colonization event; after one colonization occurs and a population locally adapts to a habitat or resource then a second colonization occurs and adapts to an unoccupied niche (Schluter 2001; Schluter et al. 2001). Alternatively, colonization may have occurred from two different source populations that brought differences that evolved in allopatry (Schluter et al. 2001).

However, in some cases, divergence may occur with gene flow in early stages (Johnson et al. 1996; Filchak et al. 2000; Johannesson 2001; Barluenga et al. 2006; Hey 2006; Savolainen et al. 2006; Bolnick and Fitzpatrick 2007; Nosil 2008). Recently colonized populations may provide ideal systems for the study of ecological divergence, as initial reproductive isolation has a disproportionate effect on divergence that may not be apparent at later stages (Coyne and Orr 2004). Very recent cases (<1000 years) of colonization and ecological divergence demonstrate that this process can occur rather quickly (Hendry et al. 2007), but cases involving populations that were not the result of introductions or manipulations by humans are rare (Diamond et al. 1989; Carroll et al. 1997). Here we present an example of ecological divergence following colonization that is both recent (∼500 years, ∼100 generations) and occurring with migration.

Sockeye salmon (*Oncorhynchus nerka*) reproduce in freshwater habitats throughout much of the North Pacific region (Burgner 1991). Several adult phenotypic traits are highly correlated with breeding environment and are believed to be the result of parallel evolution (Burgner 1991; Taylor 1991; Blair et al. 1993). Recent work has shown that adult body size and shape in sockeye are strongly related to depth and water velocity of their breeding habitat (Quinn et al. 2001). In general, sockeye males breeding along lake beaches have deeper bodies than those breeding in riverine habitats (Blair et al. 1993; Hendry et al. 2000). This appears to be a response to natural and sexual selection in the breeding environments (Quinn et al. 2001; Hamon and Foote 2005). Sockeye egg mass is correlated with breeding substrate size (Quinn et al. 1995). Because these traits are adaptations to the ecology of the site of reproduction, they may be traits that are directly responsible for reproductive isolation (Schluter 2001; Rundle and Nosil 2005).

Aniakchak National Monument and Preserve (ANMP; Fig. 1) in southwest Alaska contains the most active volcano in the Eastern Aleutian arc, having erupted more than 40 times in the last 10,000 years (Neal et al. 2001). Several of these cataclysmic geologic events are well documented and provide a framework to evaluate the timing of divergence. A massive volcanic eruption 3650 years ago formed a large caldera (Aniakchak Caldera) that filled with water forming a lake (McGimsey et al. 1994; Pearce et al. 2004). Sometime after this but before a more recent eruption that occurred 500 (standard error [SE] 369–565) years ago, the caldera wall collapsed resulting in a large flood and the formation of the Aniakchak River, which connects the caldera lake (Surprise Lake; elevation 321 m) with the Pacific Ocean through “The Gates,” a chasm breaching the caldera wall (McGimsey et al. 1994) (see Fig. 1). Sometime after this connection, sockeye salmon colonized Surprise Lake. In addition to the well-documented eruptions mentioned above, the volcano erupted again in 1931 (McGimsey et al. 1994). These eruptions probably affected breeding, rearing, and incubating conditions and may have impacted or eliminated any sockeye populations present in the caldera during that time. In fact, much of the inlet waters to the lake are presently devoid of dissolved oxygen as a result of volcanic activity (Cameron and Larson 1993) and a large portion of the associated beaches are unused by breeding sockeye. Current sockeye populations in Aniakchak Caldera may have colonized after the original ocean access following the flood, after the substantial eruption 500 years ago, or following the most recent eruption (77 years ago).

**Figure 1:**
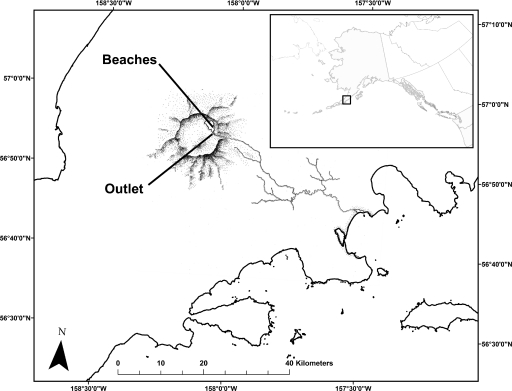
Aniakchak National Monument and Preserve (ANMP) showing Aniakchak Caldera and the two study populations.

Sockeye in Aniakchak Caldera use two breeding habitats, the outlet and the beaches, which are spatially separated by less than 1.5 km. These populations are genetically distinct (*F*_ST_= 0.01; *P* < 0.001) and together form a clade that is distinct from other populations in the area (Pavey et al. 2007). There are several different scenarios that could have resulted in this current situation. First, divergence may have occurred prior to colonization of the caldera with two different source populations; that is, a population adapted to breeding in outlets colonized the outlet and a population adapted to breeding at beaches colonized the beaches. Subsequent recent gene flow may result in convergence at neutral loci, whereas divergent ecology maintains adaptive differences. In this “two-source” model, we would expect divergence time to be considerably earlier than caldera access, perhaps >10,000 years ago when most sockeye divergence occurred following glacial retreat. In this case, the ecology and proximity of the current habitats are not informative about population divergence.

Alternatively, these populations may represent a monophyletic group that colonized the caldera and subsequently diverged in response to selection, which would yield a more recent time for divergence in comparison to the timing of colonization. This colonization prior to divergence can be considered under two scenarios that describe relatively different roles of migration in divergence (Rundle and Nosil 2005; Xie et al. 2007). In the first of these two scenarios, migration was greatly restricted through a double invasion of the habitat from a common source. In this scenario, colonization and local adaptation to one habitat occurred first, followed by a second invasion from a common source that colonized the unoccupied habitat (Schluter et al. 2001). Alternatively, in the second scenario, colonization may have occurred only once and populations diverged despite gene flow (Johannesson 2001).

In this study, we first measured ecological parameters of the breeding habitats (substrate size and rate of water flow) of these recently colonized populations. Next, we measured adult body depth and egg mass, morphological characters important to reproduction and shown to be correlated in relation to these ecological differences in many populations across the species range. Then, through applying coalescent techniques to a microsatellite database (Hey and Nielsen 2004; Won and Hey 2005), we estimated the time of onset of population divergence to see if the data suggest that the divergence took place after the availability of the habitat. Finally, we determined whether migration occurred after the onset of divergence and the relative timing of any migration events. If there is no detectible migration after divergence, the hypothesis of double colonization is supported. Migration after divergence is consistent with the hypothesis of ecological divergence despite gene flow.

## Material and Methods

### ECOLOGICAL PARAMETERS

We determined average water velocity of the outlet from cross-sectional area and previously recorded discharge (Bennett 2004). Substrate composition was determined by Wolman pebble counts (Quinn et al. 1995).

### MORPHOLOGICAL TRAITS

#### Adult body shape

We captured 301 breeding adult males by net in 2001–2003. All measurements were to the nearest millimeter. Sampling consisted of measuring midorbital to hypural length (MOH; body length) and body depth at the anterior insertion of the dorsal fin. Body depth of breeding males was compared between habitats (outlet and beach) by analysis of covariance (ANCOVA). The model included year to account for variation in overall growth and size among the different sample years, and MOH as a covariate. In addition to the measurements, we assigned spawning condition to one of three categories for each individual. We recorded males as prespawning if the fish was bright red and in good physical condition, but not expressing milt under gentle abdominal pressure. Males still in good physical condition but expressing milt were judged to be spawning, and males with extensive scarring, worn away skin, and showing a lack of slime production were categorized as senescent. We did not sample sockeye salmon showing silver coloration, as this indicates that they are still immature and their eventual spawning location and mature body shape are not finalized at that point.

#### Egg mass

Females were captured in August 2006 during spawning activity by net in the same manner as males were captured above, and MOH was measured in the same manner as for males. About 20 eggs were taken from each of 50 females at the beach habitats and 30 females at the outlet. We selected only females that were expressing eggs upon abdominal pressure. Eggs were preserved in 10% formalin. Back in the laboratory we blotted each batch of eggs with a Kimwipe (Kimberly-Clark, Dallas, TX) to remove external formalin solution. Then we measured each group of eggs to the nearest 0.1 mg. The source population of each sample was concealed during measurement. Of the 80 females for which egg samples were obtained, we eliminated 17 samples from the beach collection and six samples from the outlet collection due to connective tissue attachment. We excluded eggs with adhesions or that did not freely separate from one another. These samples may represent incomplete development so the eggs and the females that they came from were removed from the analysis. With each sample, we divided the total mass of all the eggs by the number of eggs collected to get an average mass. Egg mass was compared between habitats by ANCOVA. The model included MOH as a covariate. Body length (MOH) accounts for some variation in egg mass, so the incorporation of body length in the ANCOVA allowed us to perform residuals analysis to look at the effect of the habitat type on egg mass.

### TIME SINCE DIVERGENCE AND MIGRATION

#### Isolation with migration analysis

We performed an analysis using the program isolation with migration (IM) (Hey and Nielsen 2004) on a microsatellite DNA database from Pavey et al. (2007). We performed initial pilot runs of the program with large priors to make sure that the posterior probability area was contained within the priors. We then fine-tuned the priors to “zoom in” to show the detail of the posterior distributions while still encompassing the whole for each parameter in the model. After initial pilot runs of the program, we executed three long runs with 18 heated chains for ∼10,000,000 steps. The command line for these runs was: −q1 5 −m1 50 −m2 50 −t 1 −b 72.0 −l 24.0 −u 5 −p 4567 −n 18 −k 100 −fg −g1 0.6 −g2 0.95 −e 24.0. The first four commands set the priors for all of the parameters. The “−b” command sets the program burn in for 72 h. The “−l” command tells the program to make and output file every 24 h. The “−u” command sets the generation time of 5 years. The “−p” command sets the output options. The “−n” command sets the number of chains to 18. The “−k” command sets the number of swap attempts per step to 100. The “−fg” command sets the heating scheme to geometric. The “−g1” and “−g2” specify the degree of chain heating. The “−e” command creates a checkpoint file every 24 h. We report high point and average posterior probability estimates from all loci for time since divergence onset, migration rate in each direction, and average date of migration events. Finally, we report effective number of migrants for each population.

Because all parameters estimated in the IM model are in units of mutation, we need to estimate mutation rate to convert the parameter estimates into demographic units. Experimental work with other tetranucleotide microsatellites has demonstrated that mutation rate is often larger than the commonly used default mutation rate of 1 × 10^−4^ (Weber and Wong 1993; Ellegren 1995; Leopoldino and Pena 2003). We estimated mutation rate for each locus using two different methods. First, with the exception of *One105*, our markers are highly polymorphic, so we expect a larger than average mutation rate. We assigned the conservative mutation rate of 1 × 10^−4^ to our one moderately polymorphic marker, *One105*, and used the mutation rate scalar estimates obtained from running the IM program to estimate the mutation rates of the other loci. Second, mutation rates in tetranucleotide microsatellites are shown to vary with length of repeat unit (Leopoldino and Pena 2003). We calculated a regression equation using the data from Leopoldino and Pena (2003) which was obtained from comparing observed mutation rate with geometric mean of number of repeats. We converted estimated parameters into demographic units with the method yielding the more conservative (slower) global mutation rate, which is the geometric mean of the mutation rates scaled from the model output for all loci.

#### IM assumptions

The IM program has several assumptions about the input data. Perfect tetranucleotide microsatellites have been shown to exhibit mutations that are well described by the stepwise mutation model (Shiver et al. 1993; Leopoldino and Pena 2003), which is the model used in IM (Hey and Nielsen 2006). One marker from this database, *One110*, did not meet the requirement of the IM program of a perfect repeat and was excluded. We included the five microsatellites that followed a perfect tetranucleotide repeat pattern from this database of 268 individuals. Another assumption about the input data is that the markers should not be physically linked. We tested for linkage disequilibrium and found no significant linkage disequilibrium in any of these markers (Pavey et al. 2007). A third assumption about the input data is that the markers are not under selection. This assumption is more difficult to explicitly test. One potential indication of selection is deviations from Hardy–Weinberg equilibrium (Conner and Hartl 2004), which is not present with these data (Pavey et al. 2007) or with similar data on these same markers in another study involving sockeye salmon in southwest Alaska (Olsen et al. 2004). Another potential indication of selection is outlier loci, or one or two loci being primarily responsible for the measured genetic differences. This was not indicated with these markers in another study (Olsen et al. 2004). To determine whether this was the case in our dataset, we used the program WHICHLOCI to determine the relative contribution of each marker to the measured genetic divergence. Because we ran IM without the *One110* locus (see above) we ran WHICHLOCI both including and excluding *One110*. Also, we sequentially dropped each locus to see if this substantially affected the *F*_ST_ between these populations. We preformed this analysis in GENEPOP (Raymond and Rousset 1995).

Another assumption of IM is that the two analyzed populations are more closely related to each other than they are to other populations. (Hey and Nielsen 2006). The basic phylogenetic unit of lake-type sockeye salmon is the nursery lake (Wood 1995; Beacham et al. 2006). When glaciers recede and expose new lake habitats, sockeye colonize. Divergence also occurs among habitats within the nursery lake, but genetic differences are generally much smaller within lakes than between lakes. This is the situation at Aniakchak (Pavey et al. 2007). The *F*_ST_ between the beach and outlet populations within Surprise Lake was smaller than the *F*_ST_ between either of these populations and any other population outside of Surprise Lake. These relationships are further illustrated in our neighbor-joining tree, in which the bootstrap value for the Surprise Lake populations forming a clade received 96% support. Although the best information we have supports the hypothesis that the two Surprise Lake populations are genetically closer to each other than to other populations, genetic similarity is certainly not “proof” of common ancestry. Gene flow as well as common ancestry will result in close genetic relationships. This complication is precisely why we want to apply the IM model to this system, as it partitions these competing homogenizing processes.

As with all applications of the IM model between populations, we cannot rule out that there is some level of gene flow with other populations outside of Surprise Lake. However, due to the 300 m elevation gain that may impose a substantial migratory barrier to outside populations, as well at the close proximity and the limitation of the study to the only populations sharing Surprise lake as the nursery lake, we believe that “the history of a sample from two populations can reasonably be described by an IM model” (Hey and Nielsen 2006).

#### Migrate analysis

We ran the program MIGRATE 3.0.3 (Beerli and Felsenstein 2001; Beerli 2006) on the same dataset to compare the output with the results from IM. This model estimates similar parameters to IM, except there is no “time since divergence” parameter. The model assumes that there has been sufficient time since divergence that migration and drift subsequent to divergence has a greater effect on current genetic relationships than shared ancestry prior to divergence. We used the same mutation rates from the method above. We used the Bayesian search strategy with slice sampling Markov chain Monte Carlo (MCMC) with four heated chains. We started with experimental runs with large priors and then performed long runs with uniform priors of 0–30 for both θ and *m*. We set the burn-in for 50,000 steps and collected date for 2,400,000 steps.

## Results

### ECOLOGICAL PARAMETERS

**Figure 2:**
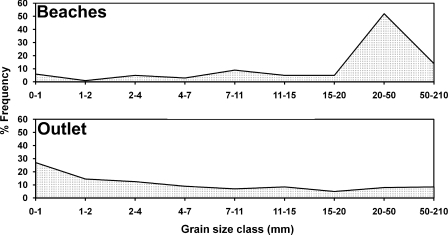
Size distribution of substrate for the two breeding locations.

Surprise Lake outlet had larger substrate than Surprise Lake beaches (Fig. 2). Surprise Lake beaches have no measurable flow in the water column, but we calculated an average current of 0.455 m/sec in the outlet.

### MORPHOLOGICAL TRAITS

**Figure 3:**
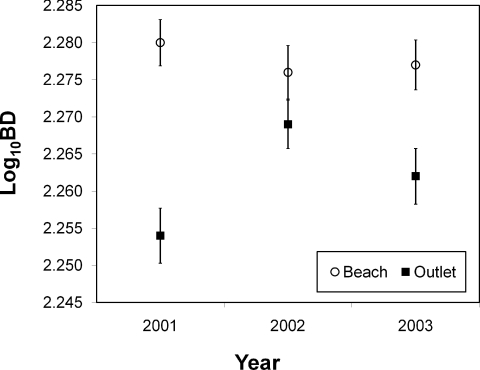
Comparison of body depth in beach and outlet males. Body depth was standardized by fitting regressions of body depth as a function of midorbital to hypural length for each population for each year. Then, the residual of body depth for each fish from the appropriate regression line was calculated, and that residual was used to calculate a standardized body depth at the average size. Beach males show consistently deeper bodies each year, though the difference is smaller in 2002.

Male MOH averaged 497.9 mm (MOH; body length) and body depth averaged 186.6 mm. There was no difference in MOH between the populations (*P*= 0.79). ANCOVA results indicated that male body depth was significantly correlated with length (*P* < 0.0001), and that both year (*P* < 0.01) and habitat (*P* < 0.001) were significant factors in determining body depth (Table 1, Fig. 3).

**Table 1:** Male body shape and female egg mass data summery. We measured midorbital to hyperal length (MOH) along with body depth (BD) in males and egg mass in females.

Sex	Year	Beach			Outlet		
		*N*	MOH (mm) (SE)	BD (mm) (SE)	*N*	MOH (mm) (SE)	BD (mm) (SE)
Males	2001	51	514.31	194.25	50	507.08	185.90
			(2.21)	(1.45)		(6.45)	(3.49)
	2002	53	500.60	191.49	50	495.00	186.00
			(5.00)	(2.86)		(7.93)	(4.10)
	2003	48	479.50	180.46	49	489.61	180.43
			(5.96)	(3.37)		(5.09)	(2.50)
	All	152	498.54	188.93	149	497.28	184.13
			(2.89)	(1.60)		(3.84)	(1.98)

	Year	*N*	MOH (mm) (SE)	Egg mass (mg) (SE)	*N*	MOH (mm) (SE)	Egg mass (mg) (SE)

Females	2006	30	468.83	99.16	22	475.36	107.38
			(3.63)	(2.65)		(4.50)	(2.78)

**Figure 4:**
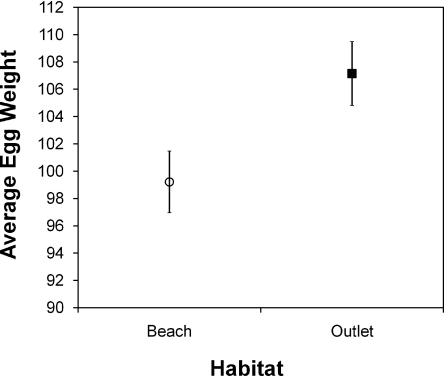
Average and standard error of standardized egg weight for beach and outlet female sockeye salmon from Surprise Lake. The residuals are taken from regressions of egg weight against midorbital to hypural length.

Egg mass was significantly correlated with MOH of females (*P* < 0.01), and habitat was a significant factor as well; the eggs of outlet sockeye were larger than those of beach sockeye (Table 1, Fig. 4; *P* < 0.02).

### TIME SINCE DIVERGENCE AND MIGRATION

Our WHICHLOCI simulation indicated that individuals could be assigned to the correct population most of the time (86.0%, SE 0.11% including *One110*; 82.3%, SE 0.12% excluding *One110*). Also, the program indicated no outlier loci, as the relative assignment ability of each locus was evenly spread (minimum score 13.4% of six loci including *One110*, 16.5% of five loci excluding *One110*). Sequential dropping of loci did not substantially change the original *F*_ST_ of 0.0112 reported in Pavey et al. (2007). The range of sequential dropping was *F*_ST_= 0.0110–0.0135.

Mutation rates calculated from the two methods were within the same order of magnitude. The geometric mean of mutation rates for all loci using the mutation rate scalar method was 7.91 × 10 ^−4^ per generation or 1.58 × 10^−4^ per year (Table 2). For the length of repeat method, the geometric mean of mutation rates of all loci was 3.40 × 10^−3^ per generation or 6.79 × 10^−4^ per year (Table 3). We used the lesser rate obtained with the scalar method for all demographic conversions.

**Table 2:** Estimated mutation rates based on mutation rate scalars.

Locus	Scalar estimate			Mutation rates	
	Average	SD	Calibrated scalar	per generation	per year
*One102*	0.9092	(0.0097)	7.0046	0.0007	0.00014
*One105*	0.1298	(0.0028)	1	0.0001	0.00002
*One108*	4.406	(0.0812)	33.9448	0.00339	0.00068
*One109*	0.7292	(0.0277)	5.6181	0.00056	0.00011
*One115*	3.0129	(0.1403)	23.2116	0.00232	0.00046
Geometric mean				0.00079	0.00016

**Table 3:** Mutation rate estimates based on length of repeat method. We include length of repeat and corresponding mutation rates for each marker.

Locus	No. of repeats	Mutation rates	
		per generation	per year
*One102*	15.22	0.00325	0.00065
*One105*	7.55	0.000085	0.000017
*One108*	18.44	0.00884	0.001768
*One109*	15.63	0.00374	0.000748
*One115*	25.69	0.0497	0.00994
Geometric means		0.003399	0.00068

**Figure 5:**
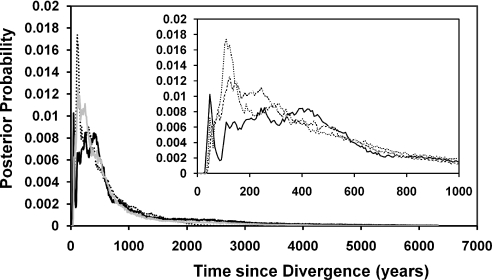
Posterior probability distributions for time since divergence in years. All three runs of the IM program are illustrated; run 1 is black dashed, run 2 is black, run 3 is gray. The inset is an enlargement of years 1–1000.

High point estimates of the posterior probability of the time since onset of divergence ranged from 47 to 123 years prior to sample collection (Table 4, Fig. 5), however, runs 2 and 3 exhibited two and three peaks, respectively, and all peaks occurred between 47 and 400 years prior to sample collection. Mean distribution values of the entire posterior probability distributions for divergence times ranged between 389 and 503 years ago (Table 4). It is important to note that with all of the posterior probabilities, the y-axis scale is completely dependent on the number of bins in the x-axis (1000). The area under the curve is equal to one.

**Table 4:** Comparison of the high point, average values, 95% credibility interval, and 90% highest posterior density (HPD) of the posterior distribution of divergence onset dates in years before sample collection for all three runs of the IM program.

	High point	Average	95% credibility interval	90% HPD
Run 1	111	389	66–1725	35–1206
Run 2	47	503	54–4358	22–2528
Run 3	123	396	73–3022	28–1560
Mean	93.7	429.3		
SD	(40.9)	(63.9)		

**Figure 6:**
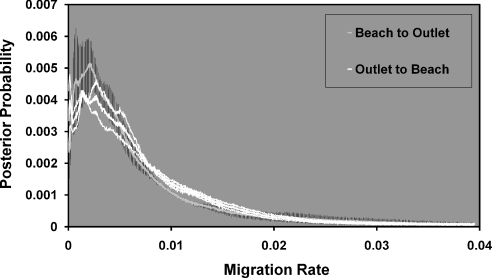
Posterior probability of migration rates between populations. The composite figure of all three runs of the migration rate posterior probability distributions shows the beach to outlet migration as a gray line on a black field. The line is the average probability for the three runs, and the black field is the standard error field. The outlet to beach average probability is the thick white line, and the standard error space around the line is depicted by the thin white lines.

**Figure 7:**
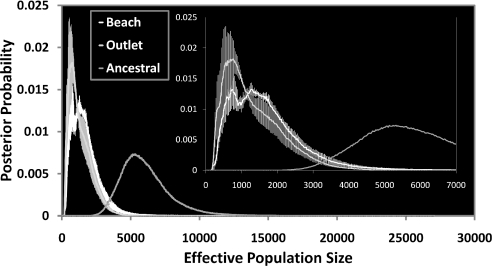
Posterior probability of effective population sizes of beach, outlet, and ancestral populations. Solid lines are the average probability for all three runs of the IM program. The fields represent the standard error of the runs for each parameter. The inset shows detail.

Migration occurred after the onset of population divergence. High point probability estimates of migration rate per generation (*m*) ranged between 0.00009875 and 0.002706 and mean probability estimates of migration rate ranged between 0.003377 and 0.006379 (Fig. 6). High point probability estimates of the average date of all migration events ranged between 42 and 120 years ago (Table 5). Mean probability estimates of the average date of all migration events ranged between 177 and 305 years ago (Table 5). High point probability estimates of the effective number of migrants (parameters θ×*m*/2) ranged between 0.16 and 9.0 (Table 6). Mean probability estimates of the posterior probability of the effective number of migrants ranged between 8.9 and 18.4 (Table 6). High point estimates of *N_E_* ranged between 740–1655 for beach, 528–988 for outlet, and 5047–5588 for ancestral (Fig. 7). Mean probability estimates of *N_E_* ranged between 1386–1709 for beach, 961–1394 for outlet, and 5561–5967 for ancestral (Fig. 7). Our results from our MIGRATE analysis were very similar to our results with IM in all the common parameters, *N_E_* and migration rate (Table 7).

**Table 5:** Comparison of the high point and average values of the posterior distribution of divergence and average migration dates in years before sample collection for all three runs of the IM program.

Direction of migration		High point	SD of five loci	Average	SD of five loci
Outlet	Run 1	44	(3.4)	177	(1.3)
to beach	Run 2	120	(31.2)	276	(2.3)
	Run 3	61	(2.8)	205	(1.7)
	Mean	74.9		219.5	
	SD	(39.7)		(51.1)	
Beach	Run 1	42	(2.8)	185	(2.8)
to outlet	Run 2	99	(28.1)	305	(6.1)
	Run 3	56	(3.5)	224	(3.3)
	Mean	66.0		238.1	
	SD	(29.7)		(60.8)	

**Table 6:** Effective migrants per generation for all three runs of the IM program. We include the high point and the average of the posterior probability distribution for both directions of migration.

Run	Outlet to beach		Beach to outlet	
	High point	Average	High point	Average
1	0.16	16.15	1.23	12.25
2	9.00	16.67	4.41	12.71
3	3.83	18.41	1.01	8.86

**Table 7:** Comparison of demographic parameters obtained with IM and MIGRATE programs, including high point, mean, and 95% credibility intervals for effective population size and migration rate for both populations.

Program	Parameter	High point	Mean	95 Low	95 High
IM run 1	*N_E_* beach	794	1384	390	3889
	*N_E_* outlet	528	961	311	3098
	*m* outlet to beach	0.00010	0.00583	0.00018	0.03115
	*m* beach to outlet	0.00024	0.00638	0.00045	0.03506
MIGRATE	*N_E_* beach	1979	2208	1417	2458
	*N_E_* outlet	1062	2147	417	1875
	*m* outlet to beach	0.00350	0.00335	0.00190	0.00439
	*m* beach to outlet	0.00622	0.00625	0.00486	0.00747

## Discussion

We have described the most recent ecological divergence reported in a fish species following natural colonization. The divergence observed here is probably in a very early stage, but the morphological differences are consistent in direction with that documented for similar ecological differences in other sockeye populations. Our results indicate that migration occurred after divergence onset suggesting that this divergence is occurring despite migration.

There are many examples of ecological divergence following natural colonization in fish including stickleback (*Gasterosteus aculeatus*; Lavin and McPhail 1985, 1993; Schluter 1996b; Rundle et al. 2000; Reusch et al. 2001), lake whitefish (*Coregonus clupeaformis*; Lu and Bernatchez 1999; Rogers et al. 2002; Derome et al. 2006), arctic char (*Salvelinus alpinus*; Gislason et al. 1999; Klemetsen et al. 2002; Johnston et al. 2004), and sockeye salmon (Blair et al. 1993; Wood and Foote 1996). However, all of these important examples are on a postglacial retreat timescale (∼10,000–15,000 years). The present study demonstrates a very recent ecological divergence following natural colonization. This ecological divergence is extremely recent (∼500 years, 100 generations), between populations of close geographic proximity (∼1500 m), and occurred despite migration.

It is possible that the actual mutation rates of the microsatellite markers used in this analysis were different than our estimate. This difference would proportionately change our converted demographic parameters: time since divergence onset, *N_E_*, average date of migration, as well as migration rate would all be affected by mutation rate. However, the relationship between divergence time and time of average migration would change similarly, making this relationship between them independent of mutation rate. The estimate for effective number of migrants is also independent of mutation rate, because mutation cancels out in the conversion process.

### MORPHOLOGY

Most sockeye salmon populations were established following glacial retreat on the order of 10,000 years ago (Wood 1995). Populations that breed in deep water along lake beaches consistently have greater average male body depth than populations breeding in flowing water environments (Blair et al. 1993). The differentiation of populations is variable, but for some populations the body depth as a function of body length is so different that there is little overlap between habitats (Hamon et al. 2000). This is particularly the case with access limiting streams or inlets and high levels of predation (Quinn et al. 2001; Hamon and Foote 2005), which is not the situation with either of the habitats in this study. Also, gill net fisheries may impose selection on body depth (Hamon et al. 2000), but the only commercial fishery on sockeye in this study is a seine fishery. Our results indicate that the male sockeye in Aniakchak Caldera are deeper bodied along the lake beaches than in the riverine outlet breeding habitat, the predicted nature of the difference based on patterns of sockeye differentiation elsewhere.

Egg size of female sockeye salmon is also differentiated among other sockeye populations since the last glaciation, with females that breed over larger substrate generally having larger eggs (Quinn et al. 1995). The substrate size along the breeding areas in Aniakchak Caldera is quite different between the beaches and the outlet river. The egg size of females in these locations has diverged in the manner that was expected; females breeding in larger substrate had larger eggs.

Both egg mass and body shape in salmon have genetic components (Gall and Huang 1988; Su et al. 1997; Kinnison et al. 2001, 2003; Gall and Neira 2004), but can also vary due to plastic responses. Outlet breeding salmon expend more energy after migration, which could lead to smaller eggs (Kinnison et al. 2001) and shallower bodies (Kinnison et al. 2003; Crossin et al. 2004). The difference in egg mass that we document here is in the opposite direction, whereas the differences in body size are consistent with energetic trade-offs. We also note that many of the environments experienced by these populations are similar. Incubating and spawning environments differ, but both populations have access to the same lake environments, migrate down the same river and the same distance to the ocean, have access to the same ocean environment, and again migrate up the same river for the same distance back to Surprise Lake. These populations have access to the same habitats, and the differences experienced are a consequence of an individual's choice, with the exception of incubation and emergence habitats, which are a consequence of an individual's parent's choice. Because we did not perform common garden rearing experiments, we cannot exclude the alternative hypothesis that phenotypic plasticity contributed to our measured morphological differences.

The observed pattern of divergence in body depth and egg size, taken together with a heritable basis for these traits established in closely related species, and the expected plastic response of egg size in the opposite direction of our measured difference, suggests some element of genetic divergence in these traits. However, the relatively small degree of divergence observed in these traits relative to other studies may have a number of causes. First, it may reflect relatively similar optimal phenotypes for the two environments in question. Second, it may result from the relatively recent divergence of these populations, and reflect that they have not yet reached the phenotypes that would be optimal for their breeding habitats. Third, it may be a plastic response that has not been previously described that is in the opposite direction as found in Kinnison et al. (2001). Fourth, selection in this case for this trait may be relatively weak. Finally, it may result from migration, and resulting gene flow, between the habitats constraining greater divergence.

### DIVERGENCE WITH MIGRATION

Our estimates obtained from the IM model indicated that divergence began recently (389–503 years; 78–100 generations ago) in a time frame that coincides with the 500 year old eruption event, and that migration occurred since (*m*= 0.003–0.006). Our IM migration and *N_E_* estimates were similarly estimated in MIGRATE. The MIGRATE 95% credibility intervals for all estimated parameters are within the bounds of the IM credibility intervals. The actual high point and mean parameter estimates are slightly higher than the IM estimates. Some differences are to be expected because MIGRATE does not have a time since divergence parameter in the model, but the overall convergence of the estimates suggest that demographic processes are more important in shaping the genetic structure than the recent common ancestry.

These results allow us to reject our first scenario of two sources that diverged long ago with an unknown geographic relationship. Also, our measurement of migration since divergence suggests that migration is present in this ongoing divergence. To assess the relative importance of allopatry and sympatry, we compared the estimates of time of divergence onset with the average time of migration (Tables 4 and 5). The time of average migration was estimated approximately midway between the estimated onset of divergence and time of sample collection in 2001–2003. This occurred in two of three runs in the comparison of high point posterior probabilities and three of three runs in the comparison of average posterior probabilities (Tables 4 and 5). These results best support the scenario that migration was present between these populations for a substantial period after divergence. However, because we do not estimate the distribution of actual migration events, only the average time of migration, we cannot compare the relative time periods of divergence with and without migration.

By applying genetic analysis techniques to a system with known temporal landmarks based on documented volcanic eruptions, we uncovered details of a case of very recent ecological divergence despite gene flow. This divergence began around 500 years or 100 generations before present. We measured migration that occurred since divergence. To our knowledge, this is the most recent ecological divergence ever reported in a fish species following natural colonization. In this case, it appears that this ecological divergence occurred despite migration.
